# Pollinator behaviour and prevalence of the anther smut *Antherospora vindobonensis* in its host, the Hungarian two-leaf squill (*Scilla vindobonensis*)

**DOI:** 10.1186/s40529-024-00437-9

**Published:** 2024-09-29

**Authors:** Pavol Prokop, Kristián Tučník, Zuzana Provazník, Zuzana Čiamporová-Zaťovičová, Fedor Čiampor Jr

**Affiliations:** 1 Department of Environmental Ecology and Landscape Management, Faculty of Natural Sciences, Comenius University, Bratislava, Slovakia; 2 Institute of Zoology, Slovak Academy of Sciences, Bratislava, Slovakia; 3 ZooLab, Department of Biodiversity and Ecology, Plant Science and Biodiversity Centre, Slovak Academy of Sciences, Bratislava, Slovakia

**Keywords:** Anther smuts, Pollinator preference, Parasite transmission, Pollen count, eDNA metabarcoding

## Abstract

Anther smuts are fungal diseases that have detrimental effects on the biology of their host plants. We investigated the transmission of Anther smut *Antherospora vindobonensis* to its host, *Scilla vindobonensis* under natural and laboratory conditions. Plants flowering early in spring were less vulnerable to disease (∼ 20–30% of plants which appeared early in the season were diseased) than flowers that bloom at the end of the season (∼ 60% of plants which appeared late in the season were diseased) which contradicts the patterns observed for other anther smuts species. Diseased plants showed thicker stems, lower nectar production and smaller flower radius than healthy plants. The disease prevalence in *S. vindobonensis* was not influenced by plant density. The production of spores in diseased flowers was 125 times higher than the production of pollen in healthy flowers. The number of pollen grains attached to pollinator bodies from healthy plants under controlled conditions was similar to the number of spores attached from diseased plants. Experienced honeybees in the field showed almost identical preferences for healthy plants (65%) over diseased ones, as did naïve bumblebees under controlled laboratory conditions (69%). eDNA metabarcoding revealed that healthy flowers attracted a significantly higher number of arthropod species (M = 6.23, SE = 1.48) than diseased flowers (M = 2.32, SE = 0.31). Strong seasonal differences in the risk of infection suggest that *S. vindobonensis* solves the trade-off between low pollinator availability and low risk of infection at the beginning of the season and high risk of infection and high pollinator availability at the end of season. By reproducing early in the season, plants can reduce the risk of disease.

**Supplementary Information:**

The online version contains supplementary material available at 10.1186/s40529-024-00437-9.

## Introduction

The coevolutionary dynamics between parasites and their hosts play a pivotal role in the functioning of ecosystems (Best et al. 2009; Buckingham and Ashby 2022). Understanding the mechanisms underlying pathogen transmission becomes crucial in predicting disease spread and host reproduction (Beldomenico and Begon 2010; VanderWaal and Ezenwa 2016). The significance of host density as a pivotal factor in driving parasite transmission in vector-borne diseases is implicitly linked to variations in host density (Keeling and Rohani 2008; Hopkins et al. 2020). Pathogen prevalence can increase with host density (Brunner et al. 2017; Ryder et al. 2005; Martí-Marco et al. 2023), but transmission can also be independent of host density (Hoyt et al. 2020, 2023). Pathogen independence from its host density works particularly when transmission is environmental, i.e. when pathogens in the environment for an extended period of time (Carver et al. 2023), or when the pathogen is transmitted by vectors or sexually (Anderson and May 1991; Antonovics et al. 1995; Thrall et al. 1993). Under certain circumstances, high parasite prevalence may lead to local host extinction (De Castro and Bolker 2005; Martin et al. 2018). However, research on the interaction between host density and vector-borne pathogens is scarce in naturally occurring populations.

Disease vectors may exhibit preferences for hosts based on their infection status, showing attraction to either diseased or healthy hosts depending on the circumstances (Sisterson 2008; Batista et al. 2014; Zeilinger and Daugherty 2014; Busula et al. 2017; Gandon 2018). For example, Ingwell et al. (2012) demonstrated that healthy aphids displayed a preference for wheat plants diseased with the barley yellow dwarf virus. Interestingly, this preference underwent a reversal once the aphids themselves became diseased. On the other hand, some species show specific preferences for healthy hosts that appear to be more attractive to vectors. Daugherty et al. (2011) revealed that grapevines diseased with the bacterial pathogen *Xyella fastidiosa* were actively discriminated by their leafhopper vector. The contrast of the behavioural responses of vectors to diseased and healthy hosts is crucial for understanding the mechanisms of disease spread.

Flower-smut fungi cause sexually transmitted diseases in plants, infecting anthers of dicots (e.g., Caryophyllaceae [*Mycrobotrium* spp.]) and monocots (e.g., Hyacinthaceae [*Antherospora* spp. syn Ustilago spp.]) by replacing pollen with infective spores (Alexander 1989; Shykoff and Kaltz 1997). Spores on flowers adhere to the pollinator’s body and are subsequently transferred to another flower, thereby spreading disease (Alexander and Antonovics 1988; Shykoff and Kaltz 1997). This suggests that disease dynamics in this system depends on pollinator behaviour (Altizer et al. 1998; Koupilová et al. 2022).

Diseased plants have altered phenology and morphology, and disease transmission can depend on host density. Plants flowering early in the season become diseased more frequently than plants flowering later (Jennersten 1988; Alexander 1989; Alexander and Antonovics 1995; Carlsson and Elmqvist 1992). Diseased plants produce more flowers likely to increase attractiveness to pollinators, or due to fungal manipulation of the host (Jennersten 1988; Alexander and Maltby 1990; Shykoff and Kaltz 1997, 1998; Carlsson and Elmqvist 1992; Shykoff et al. 1997; Verdú and Mas 2015; Bruns et al. 2017). However, some researchers showed that infection has a detrimental effect on the number of flowers and plant height, nectar production, and flower size (Baker 1947; Tojo and Nishitani 2005; Biere and Honders 1996; Jennersten 1988; Shykoff et al. 1997; Koupilová et al. 2022). Diseased plants can be less attractive to diurnal pollinators than healthy ones (Jennersten 1988; Shykoff and Bucheli 1995; Altizer et al. 1998) and increased host density is associated with greater likelihood of disease transmission (Carlsson and Elmqvist 1992; Biere and Honders 1998, 2006; Verdú and Mas 2015). The epidemiology of Anther smuts appears to be species-specific, and findings derived from one species should not be automatically extrapolated to others.

In this study, we combined several experiments on *Scilla vindobonensis* plants diseased by the *Antherospora vindobonensis* anther smut (formerly *Ustilago*) and its pollinators. Our objective was to investigate factors that determine disease prevalence over the season and pollinator responses to healthy plants and plants diseased with anther smut. First, we record changes in the prevalence of disease in plants occurring earlier and later in the season and their reproductive success and ask the following questions: (1) Are there morphological differences between healthy and diseased plants? (2) Are there any differences in infection prevalence between early and later flowering plants? (3) Is there an association between host density and disease prevalence? (4) Are there any differences in reproductive success between healthy and diseased plants? (5) How many pollen grains produce healthy and diseased plants and how many spores are produced by diseased plants? Second, we investigate the pollinator preferences for healthy and diseased plants. We asked the following questions: (1) To what extent do pollinators avoid visiting diseased over healthy plants in the field and in the laboratory? (2) Do pollinators make shorter visits to diseased plants than to healthy plants? (3) How many pollen grains and spores do pollinators carry after a single flower visit under laboratory conditions? We performed our experiments under natural conditions, where these interactions were documented more than 100 years ago (Bäumler 1890), and complementary data were collected under controlled laboratory conditions. We predicted that diseased plants are smaller than healthy plants, infection prevalence is higher at the beginning of blooming season, host density is positively associated with disease prevalence, and that the reproductive success of the host is compromised by disease. Furthermore, we hypothesized that pollinators prefer healthy flowers over diseased flowers and that their visits to diseased plants are shorter than visits to healthy ones. Finally, we predict that the production of spores by diseased hosts is higher than the production of pollen grains by healthy plants.

## Methods

### Prevalence of diseases and reproductive success of host plants in the field

**Fig. 1:**
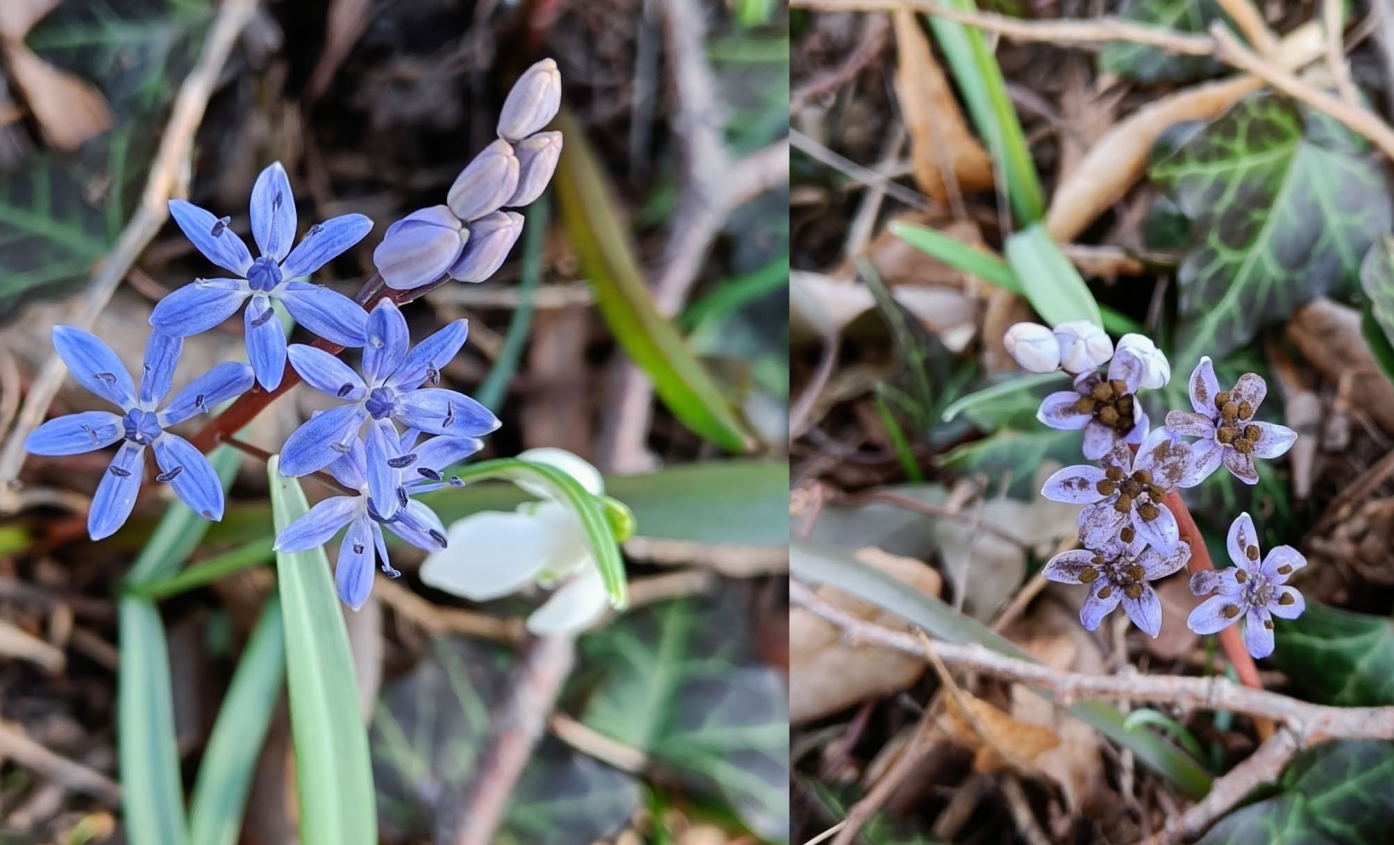
Healthy (left) and diseased *S. vindobonensis* (right) in the study area

The research was carried out in a protected area ʺJarovská Bažanticaʺ. The deciduous forest, covering an area of 78 hectares, is situated near Bratislava, with coordinates 48°04′48″N 17°05′27″E). We have been visiting this site since February 2023. On March 3, 2023, we randomly selected 15 plots (0.5 × 0.5 m squared) when the two-leaf squill (Scilla vindobonensis) was in bloom. All individuals of *S. vindobonensis* were uniquely marked with a ribbon. The flowering period of individual *S. vindobonensis* flowers typically lasts for approximately one month. We recorded the health status of each plant according to the presence of fungal spores on the flowers (diseased or not, Fig. 1), the height of the stem (± 1 cm) and the number of flowers in the inflorescence. The transects were inspected each week and new individuals of *S. vindobonensis* were marked and examined. All individuals were repeatedly inspected during the flowering season, and symptoms of disease never appeared on originally healthy plants. Similarly, diseased plants remained diseased throughout the flowering season. Data from five transects are incomplete because they were damaged by forest workers during our research. In April, when the flowering of S. *vindobonensis* ceased, we inspected the plots two times for the presence of seeds.

### Morphological differences between healthy and diseased host plants

All *S. vindobonensis* plants (*N* = 396) were removed from a randomly selected plot (10 × 10 m) on one occasion at the beginning of March and transported to the laboratory to examine possible morphological differences between healthy and diseased individuals. In the laboratory, we separated healthy and diseased individuals to examine the total prevalence of the disease and then measured the diameter and thickness of the flower stems 1 cm below the inflorescence using a digital calliper with 0.01 mm accuracy. Only one randomly selected open flower per inflorescence was chosen to examine the diameter of the flower. The height was measured with ruler to the nearest 1 mm. To examine the total number of pollen grains and spores, we randomly selected unopened flower buds from healthy (*N* = 23) and diseased individuals (*N* = 23) and stored them individually in Eppendorf tubes with 70% ethanol. Only one bud from one plant was selected.

### Nectar production

We examined nectar production by measuring the volume of nectar present in one randomly selected flower per inflorescence. We bagged healthy (*N* = 19) and diseased plants (*N* = 14) for 24 h prior to sampling between 12:00 and 13:00 h at the end of February 2023. The nectar was collected in microcapillary tubes (Hirschmann Laborgeräte, Eberstadt, Germany). The nectar volume was calculated from the height of the nectar column.

### Flower preferences of pollinators in the field

We visually tracked honeybees (*Apis mellifera*) as the most common pollinators of *S. vindobonensis* (unpublished data) in March 2023 during a sunny, windless day with maximum temperature 16.4 °C. Once a flying bee was found, it was tracked until it sat in an inflorescence of *S. vindobonensis.* We recorded whether the preferred flower was healthy or diseased and how much time the bee spent in the inflorescence. After each record, we changed location for about 10 m to minimise repeated observations of the same bee. A total of *N* = 96 records were made between 11:00 and 14:00 when pollinator activity was highest.

### Flower preferences by pollinators in the laboratory

We used bumblebees (*Bombus terrestris*, L.) as model pollinators to examine the preferences of bees for visually contaminated and healthy plants. A captive colony of naïve bees was obtained from Koppert© (Nové Zámky, Slovakia) and were kept at 22–24 °C in a room lit with natural light and neon light (370 lx). The bees were connected to a 90 × 50 × 40 cm insectarium using a plastic mesh tube and daily fed exclusively with honey solution (water 60% and honey 40%). We avoided pollen feeding to prevent bee contamination with pollen grains to ensure that all *A. scillae* spores and flower pollen grains in their bodies are the product of experimental conditions. The bees were individually tested in the insectarium. The trials started with insertion, placing two freshly collected two-leaf squill plants in glass test tubes on the front of the terrarium, 5 cm away from the back wall and 10 cm apart from each other. We standardised the number of flowers per each plant (healthy and contaminated) to *N* = 5 flowers per test plant. On a given day, the placement of the flowers was randomly determined (i.e., left or right). When the bee was feeding inside the flower for 5 s, it was quickly removed and fixed in 70% ethanol for further examination of the pollen loads (see below). Each flower and each bee were used only once. The trials took place 1 week after the colony arrived, in March 2023. All trials (*N* = 40) took place between 09:00 AM and 14:00 PM.

### Pollen/spores removal from bee bodies

The bees were stored in Eppendorf^®^ microcentrifuge tubes with 70% ethanol and removed in October 2023. Samples were vortexed for 10 min with Heidolph Reax Top and subsequently filtered through a filter paper (Fisher Scientific Filter Paper F13A 125 mm). Filter papers with pollen grains/spores were placed in a laboratory dryer (Memmert UF30) at 60 °C for 10 min until dry. Pollen grains/spores were removed from the surface of the filter paper by swabbing them with a cube (approximately 4 × 4 × mm square) of Fuchsin jelly (Kearns and Inouye 1993) using a clean entomological pin. The jelly was then placed on a slide glass and placed in the laboratory dryer. After the jelly was melted, the drop (liquid jelly) was covered with a coverslide. Pollen grains or spores were immediately captured with a cell phone (Xiaomi Redmi Note 10PRO) under the LEICA DM 200LED (40× total magnification). The images were then transferred to the computer and counted.

### Pollen and spores count in the flower host

From diseased and healthy plants, we collected and stored 23 unopened buds per plant, with a total of 46 buds (23 from diseased and 23 from healthy plants), in 90% ethanol. Pollen and spores were released from the anthers by sonication in an ultrasound bath (Bandelin RK 31) for 10 min. The solution was then evaporated from the tubes at 60 °C in the laboratory dryer (Memmert UF30). The dried pollen grains were resuspended in 1 ml of 70% ethanol: glycerol (4:1) solution and again sonicated. 10 µl of the solution in three replicates were placed on a glass slide with a micropipette, covered with cover glass and all pollen grains were counted. The total amount of pollen grains and spores left in a flower was therefore calculated by multiplying the average pollen grain count in a 10 µl volume by 100. The mean values of three counts were used for statistical analyses.

### DNA metabarcoding

Randomly selected healthy (*N* = 22) and diseased (*N* = 17) flowers were collected on March 17, 2023 at “Jarovská Bažantnica”. Flower samples were stored in sterile plastic bags at -20 °C until processing. DNA was extracted from all diseased and all healthy flowers. The extraction and purification of genomic DNA were done using the DNeasy blood & tissue kit (Qiagen), following manufacturer’s protocol. PCRs were conducted with 2 replicates per extract and 6 negative controls. A 418 bp fragment of the mitochondrial cytochrome c oxidase subunit 1 (COI) gene was targeted in a two-step PCR (Elbrecht and Steinke 2019). In PCR1, the fragment was amplified from 1 µL of DNA template, using basic BF3/BR2 primers (Elbrecht et al. 2019) (0.4 µM each), AccuStart™ II PCR ToughMix and GelTrack Loading Dye (1x, Quantabio), filled to a total volume of 10 µL with nuclease-free water (Sigma-Aldrich). The thermal cycling program started at 94 °C for 5 min, proceeding with 25 cycles of 94 °C for 30 s, 50 °C for 30 s, 70 °C for 50 s, and a final elongation at 70 °C for 10 min. Similar conditions were used for PCR2, except that the number of cycles was decreased to 18 with elongation adjusted to 65 °C for 2 min per cycle. Each well acquired unique combination of tagged BF3/BR2 fusion primers (Elbrecht and Steinke 2019) in PCR2, and 1 µL of respective PCR1 product served as DNA template. The PCR2 products were pooled equimolarly based on fluorometric quantification (Quantus™ Fluorometer, QuantiFluor^®^ ONE dsDNA System, Promega) and purified using 0.8x SPRIselect beads (Beckman Coulter). The final 15 pM sequencing library included 10% PhiX, and was analyzed on Illumina MiSeq with Reagent Kit v3, 2 × 300 bp at the Institute of Chemistry, Slovak Academy of Sciences. Raw sequence data was filtered and processed in the mBrave application (www.mbrave.net), in which determination also took place (i.e., blasting against the reference barcode database BOLD – Barcode of Life Data Systems, www.v4.boldsystems.org). To increase the confidence of subsequent analyses, operational taxonomic units (OTUs) represented by < 5 reads were removed from the resulting list, subsequently using only insect OTUs (putative species) that are apparently related to flowers and therefore their visit was highly probable.

### Statistical analyses

The prevalence of infection in the field (coded as diseased = 1, healthy = 0) was defined as a dependent binomial variable in the Generalised Mixed Model (GMM). The time of season (categorical predictor) was subjectively divided into early flowering plants (before 8. March 2023) and late flowering plants (after 8. March 2023). The 8th of March was chosen as a subjective midpoint to divide the flowering season into early and late flowering periods, considering that *Scilla* starts flowering by the end of February and no other plants appear after 20th March. Plant density and number of flowers per plant were continuous predictors and transect ID was treated as a random effect. Because the height of the plant was moderately correlated with the total number of flowers (Pearson *r* = 0.48, *P* < 0.001, *N* = 156), we did not simultaneously include these two variables in the model to avoid multicollinearity. Instead, we tested each variable separately. Note that even the inclusion of both the height of the plant and the number of flowers did not influence the results of GMM. Similarly, the influence of plant density and number of flowers (independent variables) on plant reproductive success (binomial dependent variable, capsule absent = 0, capsule present = 1) was examined with the same procedure. Pollinator preferences of diseased and healthy plants in the field as well as in the laboratory were examined with the binomial test. The time the honeybee spent on the flower in the field was recorded to the nearest of 1 s. Data were not normally distributed (Shapiro-Wilk test, W < 0.887, *P* < 0.001), and a comparison of time spent in diseased and healthy flowers was performed using the Mann-Whitney (M-W) U test.

Nectar production data from diseased flowers were not normally distributed (Shapiro-Wilk test, W = 0.58, *P* < 0.001) and were compared using the M-W U test. The mean number of pollen grains and spores showed a normal distribution (Shapiro-Wilk test, W > 0.9, *P* > 0.15). The pollen grains against the spores were compared with a t-test for independent samples. The mean number of insect taxa between diseased and healthy plants detected with DNA metabarcoding was compared with the Generalized Linear Model (GLM) with Poisson distribution of the dependent variable.

## Results

### Plant morphology and infection

Diseased plants were significantly thicker and showed a smaller flower radius than healthy plants. There were no differences in plant height between the two groups (Table 1). Healthy plants produced significantly higher volumes of nectar (mean = 0.53 µl, range: 0.1–1.3 µl, SE = 0.08) than diseased flowers (mean = 0.1 µl, range: 0–0.7 µl, SE = 0.04) (MW U = 22.0, *P* < 0.001).

**Table 1:** Differences in morphology between healthy and infected plants. Values are means ± SE. Sample sizes are in parentheses

	Plant height (cm)	Stem thickness (mm)	Flower diameter (mm)
Healthy	13.8 ± 0.45 (47)	1.79 ± 0.05 (74)	1.69 ± 0.03 (47)
Diseased	13.46 ± 0.49 (50)	2.14 ± 0.05 (77)	1.54 ± 0.02 (50)
*t*	0.51	4.72	4.57
df	95	149	95
*P*	0.61	< 0.001	< 0.001

### Predictors of infection in the field

Out of *N* = 396 individuals of the two-leaf squill collected at the beginning of the blooming season, *N* = 83 (21%) were visually diseased by *A. scillae*. These data were collected from a single census and were not compared with data from the end of the season. Considering diseased plants on *N* = 15 plots, the percentage of diseased plants varied between 0 and 61.53% (mean = 32.71%, SE = 4.59). The overall density of the plants within the experimental plots also varied considerably (mean = 10.66, range: 5–18, SE = 0.99, *N* = 15). GMM based on *N* = 145 plants (the data from two transects were omitted due to damage by forest workers) showed that the probability of infection was significantly influenced by the time of season (Table 2). Although only 29/97 (30%) plants were diseased at the beginning of the blooming season, 29/48 (60%) were diseased at the end of the blooming season. Higher plant density tended to be associated with a higher prevalence of disease, but the effect was not significant.

**Table 2:** Results of GMM on prevalence of infection

	Estimate	χ^2^	df	*P*
Number of flowers	0.00744	0.00884	1	0.925
Plant density	0.09894	3.27745	1	0.07
Time of season	1.38684	13.169	1	< 0.001

There were no differences in the total number of flowers per inflorescence between diseased (M = 4.41, SE = 0.33, *N* = 58) and healthy plants (M = 4.29, SE = 0.22, *N* = 87). The inclusion of plant height in the model did not significantly affect these results and its influence was not significant (Table 2).

### Impact of infection on the host’s reproductive success

The sample sizes for the reproductive success are smaller than the original samples (*N* = 84), because some transects were destroyed during the season and some plants together with ribbons were completely missing for unknown reasons. It was apparent that most of the healthy plants (34/50, 68%) produced at least one capsule, while only one of 34 diseased plants did so (2.9%). GMM showed that infection exclusively predicted the reduced reproductive success of the host plant (Table 3), whereas plant density and number of flowers did not. The time of season (or an interaction with infection) did not influence the reproductive success of *S. vindobonensis*. A total of 49% of plants (23/47) reproduced successfully early in the season, while 32.4% (12/37) did so late in the season. The inclusion of plant height in the model did not significantly affect these results and its influence was not significant.

**Table 3:** GMM on the occurrence of capsules in *S. Vindobonensis*

	Estimate	χ^2^	df	*P*
Time of season	0.521	0.597	1	0.44
Infection	-4.47	16.109	1	< 0.001
Number of flowers	-0.127	0.887	1	0.346
Plant density	0.103	0.996	1	0.318

### Impact of infection on pollinator preferences

A total of the 62 of 96 honeybees (65%) observed in the field preferred healthy plants over diseased plants (binomial test, *P* < 0.001). Pollinators spent a similar amount of time interacting with both diseased plants (mean = 9.82 s., SE = 1.60, *N* = 34) and healthy plants (mean = 10.84 s., SE = 1.19, *N* = 62) (MW U test, U = 889.00, *P* = 0.21). Out of 40 choice trials in the laboratory, one was excluded, because the bee quickly moved from one flower to another. Of the remaining 39 trials, 27 bees (69%) preferred healthy plants over diseased plants (binomial test, *P* < 0.001).

### Pollen and spores removed from bees

Four samples were damaged and could not be included in the analysis: three from healthy flowers and one from diseased flowers. The mean number of pollen grains (M = 82.6, range: 3–620, SE = 29.8, *N* = 24) and spores (M = 22.6, range: 3–56, SE = 5.42, *N* = 11) from bees visiting healthy and diseased flowers in the laboratory did not differ significantly (MW U test, U = 115, *P* = 0.56), respectively. Interestingly, while bees did not transport any spores from healthy flowers, diseased flowers contained both spores (mean values are shown above) and pollen grains. The percentage of pollen, calculated from pooled data derived from pollen grains and spores collected by bees visiting diseased flowers, ranged from 0 to 62.5% (Mean = 11.1, SE = 18.4, *N* = 11). The diseased flowers were probably contaminated by pollen transferred by pollinators in the field. Alternatively, the pollen found on diseased flowers may be residual pollen from the original plants, which were likely not wholly castrated.

### Pollen and spores count in the flower host

**Fig. 2:**
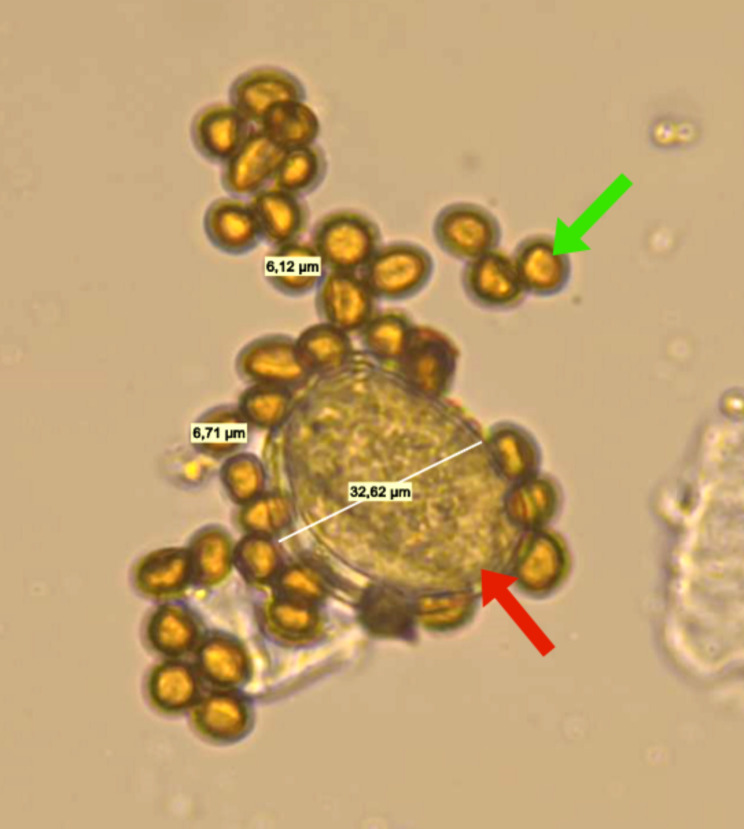
Differences in the size of *S. vindobonensis* pollen grain (red arrow) and *A. vindobonensis* spores (green arrow)

The mean number of pollen grains in healthy flowers was 5621 ± 839 (range: 475–16600, *N* = 23). The mean number of spores in diseased flowers was 703,913 ± 57,418 (range: 206867–1510733, *N* = 23). The differences between the mean number of pollen grains and spores were significant (t = 12.16, df = 44, *P* < 0.001). None of the healthy flowers contained spores, but two diseased flowers contained one pollen grain per flower. Pollen grains were typically larger than fungal spores (Fig. 2).

### Number of species detected by DNA metabarcoding

**Fig. 3:**
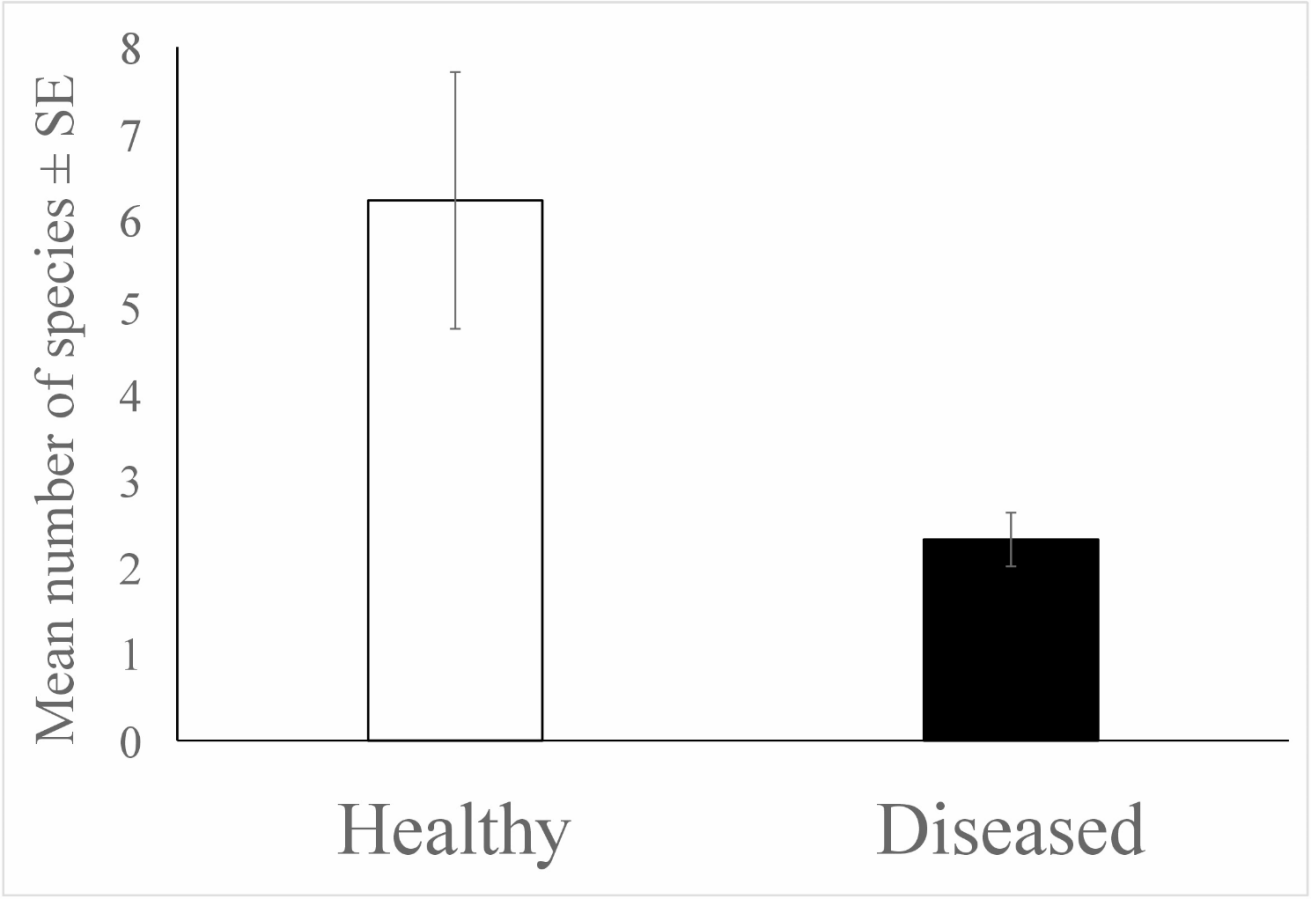
Differences in mean number of insect species detected on healthy and diseased flowers

The environmental DNA analysis of Scilla flowers revealed 51 insect operational taxonomic units (OTUs) identified from healthy flowers. Six species were found in both the healthy and diseased groups. Additionally, 24 OTUs were detected in the diseased flowers, with 8 of these OTUs also present in the healthy group. Concerning individual samples, 1–24 OTUs were recorded for healthy flowers, while only 1–4 OTUs for those diseased by smut. Healthy flowers attracted a significantly higher number of insect species compared to diseased flowers (Fig. 3). Full list of insect species identified by eDNA metabarcoding is shown in Appendix.

## Discussion

Our main goal was to study the dynamics of anther smut disease *A. vindobonensis* parasitising *S. vindobonensis* and host preferences by disease vectors. We found that disease significantly influenced certain aspects of host morphology and its prevalence was influenced by the time of season. The disease had a sterilising effect on flower reproductive organs (no pollen in the anthers, ovary abortion), and diseased plants were significantly less attractive to their potential pollinators, which transmit spores to other plants. The latter result was confirmed by field observations, behavioural experiments in the laboratory, and DNA metabarcoding analyses.

The diseased plants appeared to have thicker stems and smaller flowers, but their height was similar to that of healthy plants. There were no differences in the total number of flowers per inflorescence between diseased and healthy plants. Smuts show different effects on host biology. Our results do not support reports on detrimental effects of the parasite on number of flowers or plant height (Tojo and Nishitani 2005; Biere and Honders 1996). However, the infection had a negative influence on the flower radius, similar to the effects reported for *Ustilago violacea* parasitizing *Silene* species (Alexander and Maltby 1990; Biere and Honders 1996).

Diseased flowers offered lower nectar and no pollen rewards in our research, similarly as many other smut species (see, e.g., Jennersten 1988; Shykoff et al. 1997; Koupilová et al. 2022). It appears that fungal disease increases host attractiveness by changing stem thickness, which is typical for robust individuals that can be more attractive to pollinators (Alexander and Maltby 1990; Shykoff and Kaltz 1997, 1998). This explanation appears unlikely for diseased *S. vindobonensis*, as neither plant height nor flower number was significantly greater in infected plants. Alternatively, more attractive, robust plants can be more frequently visited by pollinators and, therefore, diseased by the greater likelihood than less robust plants (Alexander and Antonovics 1988; Alexander 1989). The expanded leaf blades of sugarcane plantlets diseased with *Ustilago scitaminea* sound similarly (Singh et al. 2004), so we assume that thick stems are consequences of infection rather than a preference for attractive flowers by pollinators. However, these alternatives are still discussed (Bruns et al. 2017) and require further research.

Most previous research showed that the prevalence of infection is highest at the beginning of blooming season (Jennersten 1988; Alexander 1989; Alexander and Antonovics 1995; Carlsson and Elmqvist 1992). It was hypothesized that a seasonal shift in disease prevalence could facilitate disease transmission. Naive pollinators initially visiting both diseased and healthy flowers may later prefer healthy flowers as they learn to distinguish between them, thus contributing to the spread of disease (Jennersten 1988). Our research showed an opposite pattern, providing no support for Jennersten’s hypothesis. We speculate that host-parasite co-evolutionary arms races favour early flowering plants that are less diseased, because low temperatures at the beginning of season are associated with low pollinator abundance. However, early flowering in spring is costly, as it results in reduced seed production due to inadequate pollination services (Kudo and Ida 2013; Kehrberger and Holzschuh 2019). Our study did not show a direct effect of the time of season on the reproductive success of *S. vindobonensis*. However, the prevalence of infection covaries with the time of the season, because the time of the season has an indirect effect on reproductive success through its influence on infection occurrence. Therefore, it appears that *S. vidobonensis* resolves the trade-off between the risk of disease transmission late in the season and the low abundance of pollinators at the beginning of the season, both of which can affect plant reproductive success. We hypothesize that early flowering plants may have a reproductive advantage due to low disease prevalence and reduced competition for pollinators in cooler spring temperatures. Conversely, late flowering plants may benefit from increased pollinator abundance as temperatures rise later in the season.

It is unclear whether early flowering is a fixed genetic trait in *S. vindobonensis*, or if it varies based on environmental factors like temperature and photoperiod. If early flowering is genetically determined, plants that bloom early each season could have inherent resistance or escape disease by flowering before pathogens/disease vectors are abundant (Alexander and Antonovics 1995). Suppose early flowering is a plastic response to environmental cues. In that case, the plants that bloom early in a given year may not necessarily be the same individuals that flower early the following season. Research on *Silene alba* infected by the anther smut *Ustilago violacea* has found negative genetic correlations between early flowering and disease resistance (Aexander & Antonovics 1995). This suggests a potential trade-off, where selection for early flowering could simultaneously select for disease susceptibility, or vice versa. There may be a “cost of resistance” - plants that flower early may have higher fitness in the absence of disease, but this could come at the expense of increased vulnerability to pathogens (Alexander and Antonovics 1995).

Plant density did not influence likelihood of infection which on the first look contradists with other works on parasite transmission (e.g., Carlsson and Elmqvist 1992; Biere and Honders 1998, 2006; Verdú and Mas 2015; Martí-Marco et al. 2023). However, our field work was carried out in a single wood, which is relatively isolated from other fields by agricultural land. Before drawing firm conclusions, it is recommended to conduct additional research that includes a wider range of localities with varying density of *S. vindobonensis.* Second, it is possible that due to the high prevalence of disease in the studied population (60% by the end of the season), the variability of infection in more and less dense microhabitats is similar, and this is why the results were independent from host plant density.

Diseased and sterilised plants, on average, produced 125 times more spores per flower than healthy plants produced pollen grains. Despite this large difference, pollen was deposited on pollinator bodies at rates like those of *A. vindobonensis* spores. These results should be viewed with caution, as the interactions between pollinators and *S. vindobonensis* in the laboratory were artificially limited to 5 s to standardize experimental conditions, whereas natural interactions between *S. vindobonensis* flowers and pollinators typically last longer. However, the overabundance of spores finally overcomes any barriers to transmission. For instance, Alexander and Maltby (1990) found that although only 25% of *Silene alba* hosts were diseased, almost all (97%) of healthy plants had deposited spores of *Ustilago violacea* smut on their flowers during peak flowering. Extreme spore production counters lower transmission success and pollinator visitation rates.

In theory, pollinators should visit both healthy and diseased flowers to successfully spread infection (Real et al. 1992). This is the reason why we did not observe an extremely strong bias toward preference for healthy flowers, which corresponds to other authors studying different host species (Jenersten 1988; Carlsson-Granér et al. 1998; Koupilová et al. 2022). Given that the preferences of experienced honeybees in the field were almost identical with the preferences of bumblebees under controlled laboratory conditions, we assume that the experience of the disease did not play a role in the preferences of healthy flowers. Plant odour and colour (Dobson & Bergstrom 2000; Chittka 2022) are better candidates for slight but significant avoidance of diseased flowers. Our future research goals include investigating the reflectance spectra of both diseased and healthy *S. vindobonensis* flowers.

Alternatively, wild honeybees were similarly inexperienced as bumblebees in the laboratory, simply because their short lifespan prevented them from having any prior experiences with S. vindobonensis from the previous year. This lack of experience could explain why there were no differences in visit time between diseased and healthy plants in the field. For instance, Koupilová et al. (2022) showed that wild pollinators left diseased Dianthus carthusianorum plants after shorter visits compared to healthy plants. However, D. carthusianorum flowers between June and September, meaning that pollinators could generalize their experiences from visiting different diseased plant species during this extended flowering period. In contrast, *S. vindobonensis* has a much shorter flowering window, and its flowering is not preceded by other species, limiting the ability of pollinators to learn and discriminate between diseased and healthy plants of this species.

Pollen grains were five times larger than fungal spores. The smaller size of A. vindobonensis spores allows for greater abundance in the anthers of the host plant, *S. vindobonensis*. As a result, more spores can fit within the anther structure, increasing the chances of infection (Piątek et al. 2011). We also speculate that the smaller size of *A. vindobonensis* spores also facilitates their transfer by insect vectors. Insects can carry more spores due to their smaller size, which enhances disease prevalence.

Because diseased flowers were visited by various arthropods identified with eDNA metabarcoding less frequently than healthy flowers, it appears that insects detect the presence of infection well. Different preferences of diseased flowers by nocturnal pollinators (see, e.g., Real et al. 1992; Altizer et al. 1998) can be ruled out for our study population, because nocturnal temperatures during the blooming season of *S. vidobonensis* are low (around zero and less). eDNA metabarcoding determined only two species of diurnal moths (*Aphelia paleana*, *Eana incanana*) further supporting our suspicion that *Scilla* in our study area is pollinated exclusively by diurnal pollinators. Most species, however, likely interact with the flowers of *S. vindobonensis* for purposes other than pollination. Some dipterans, such as syrphid flies (Dunn et al. 2020), *Empis livida* (Burkill 1946), and *Chloromyia formosa* (Gibson et al. 2006), could be considered as potential pollinators of *S. vindobonensis*. Meanwhile, some species, such as hymenopterans from the family Tenthredinidae, may accidentally provide pollinator services.

Unfortunately, the presence of honeybee, as our team most frequently seen pollinator of *Scilla*, was not confirmed by eDNA metabarcoding. Considering that approximately 30–50% of *Scilla* individuals do not produce seeds (P. Prokop, personal observations; this study), it appears that in an environment characterized by intense competition for pollinators, our sample size may not have been adequate. Our independent experiments with eDNA metabarcoding on different species revealed honeybee DNA, thus we are convinced that this method is reliable.

In conclusion, seasonal time, rather than plant density, has a significant impact on the prevalence of disease in *S. vindobonensis*, which serves as the host for the anther smut *A. vindobonensis*. Early flowering plants appear to be less vulnerable to infection than late flowering plants because pollinators are scarce at the beginning of the blooming season. Using both experienced (honeybees) and naive (bumblebees) pollinators, we showed that both species prefer healthy over diseased plants. Disease did not influence the height and number of flowers in host plants; therefore, these morphological parameters could not influence host attractiveness for pollinators. Instead, the overproduction of spores can mitigate the lower attractiveness of diseased plants to pollinators. More research is needed to investigate pollinator behaviour and prevalence of disease during the blooming season, when the availability of healthy plants drops down.

## Electronic supplementary material

Below is the link to the electronic supplementary material.

Appendix: List of species visiting *S. vindobonensis* identified by eDNA metabarcoding

## Data Availability

Data are available in corresponding author upon request.
